# Software pipelines for RNA-Seq, ChIP-Seq and germline variant calling analyses in common workflow language (CWL)

**DOI:** 10.3389/fbinf.2023.1275593

**Published:** 2023-11-07

**Authors:** Konstantinos A. Kyritsis, Nikolaos Pechlivanis, Fotis Psomopoulos

**Affiliations:** ^1^ Institute of Applied Biosciences (INAB), Centre for Research and Technology Hellas (CERTH), Thessaloniki, Greece; ^2^ Department of Genetics, Development and Molecular Biology, School of Biology, Aristotle University of Thessaloniki, Thessaloniki, Greece

**Keywords:** genomics, transcriptomics, epigenomics, workflow language, containers, workflow automation, reproducibility, reusability

## Abstract

**Background:** Automating data analysis pipelines is a key requirement to ensure reproducibility of results, especially when dealing with large volumes of data. Here we assembled automated pipelines for the analysis of High-throughput Sequencing (HTS) data originating from RNA-Seq, ChIP-Seq and Germline variant calling experiments. We implemented these workflows in Common workflow language (CWL) and evaluated their performance by: i) reproducing the results of two previously published studies on Chronic Lymphocytic Leukemia (CLL), and ii) analyzing whole genome sequencing data from four Genome in a Bottle Consortium (GIAB) samples, comparing the detected variants against their respective golden standard truth sets.

**Findings:** We demonstrated that CWL-implemented workflows clearly achieved high accuracy in reproducing previously published results, discovering significant biomarkers and detecting germline SNP and small INDEL variants.

**Conclusion:** CWL pipelines are characterized by reproducibility and reusability; combined with containerization, they provide the ability to overcome issues of software incompatibility and laborious configuration requirements. In addition, they are flexible and can be used immediately or adapted to the specific needs of an experiment or study. The CWL-based workflows developed in this study, along with version information for all software tools, are publicly available on GitHub (https://github.com/BiodataAnalysisGroup/CWL_HTS_pipelines) under the MIT License. They are suitable for the analysis of short-read (such as Illumina-based) data and constitute an open resource that can facilitate automation, reproducibility and cross-platform compatibility for standard bioinformatic analyses.

## Background

Over the past 2 decades, computational analysis has become a key component of research, and especially in domains generating big data such as life sciences. Reduction in costs, and advancements in high-throughput methods and technologies resulted in the generation of large amounts of data from different omics fields, such as genomics, epigenomics and transcriptomics (Muir et al., 2016). Many of these biological datasets are accessible to the scientific community through databases, such as the European Nucleotide Archive (ENA) (Leinonen et al., 2011a) and the Sequence Read Archive (SRA) (Leinonen et al., 2011b), or large consortia, such as the 1,000 Genomes (Auton et al., 2015) and The Cancer Genome Atlas (TCGA) (Weinstein et al., 2013), with their ease of access leading to significant discoveries. This plethora of large-scale and diverse omics data is primarily driven by the understanding that a single -omics type provides only a limited view of the underlying complex biological mechanisms to gain sufficiently meaningful insights. Therapeutic interventions in the complex pathologies evident in common diseases, including cancer or neurodegenerative disorders, require a systems-approach that aspire to: i) unravel salient biological motifs coordinated by several molecules, ii) deduce which changes between disease and control groups are causative or simply a subsequent product of the disease, and iii) reliably identify potential biomarkers and “druggable” targets (Hasin et al., 2017; van Hasselt and Iyengar, 2019).

Reflecting the complexity of the bulk of High-throughput Sequencing (HTS) generated data, the corresponding software tools required for processing and analyzing them are equally advanced; they often come with dependencies, such as requirements for pre-installed software, expect a specific order of execution within a larger workflow, and usually custom scripts are required to integrate them into functional pipelines. Therefore, a considerable amount of time and effort is dedicated to creating suitable computational environments, where software pipelines aiming to analyze HTS data can be executed. Moreover, it is quite common for software pipelines to be designed to serve the needs of a specific study or experiment solely, therefore extensively incorporating custom scripts, which are rarely designed with sustainability in mind, and even including the occasional manual step, such as manual file conversion to achieve format compatibility. However, these practices should be avoided given that they: i) are inefficient and error-prone, ii) hinder the reproducibility and reusability of the workflow, and iii) lead to the need for further time-consuming and laborious development in order to be reapplied (Sandve et al., 2013).

It is now well known that reproducing published results and reapplying peer-reviewed and publicly available software tools in new experiments has become challenging and/or error-prone (Baker, 2016; Collberg and Proebsting, 2016; Stupple et al., 2019), with global efforts and initiatives explicitly targeting this. Reproducibility and reusability, although often neglected, are critical factors that computational biology tools and workflows are called to satisfy when applied to biological research. Ideally, scientific results should be reproducible, both by the original party that performed the analysis and third-party peers, and software pipelines should strive to be reusable for the analysis of novel data. These characteristics increase productivity, build trust and encourage the wider scientific community to adopt a particular software tool.

A solution for mitigating issues on reproducibility and reusability is the utilization of formal workflow languages and standards, such as the Common Workflow Language (CWL), an open standard describing and implementing the creation of data analysis workflows (Chapman et al., 2016). A major advantage of CWL is that it is platform-independent, meaning that CWL workflows can be executed on any platform, be it a local machine, a cluster, or a cloud-based infrastructure. This makes CWL workflows highly portable and allows for seamless execution across different computing environments. Furthermore, CWL is designed to be interoperable with other bioinformatics tools and platforms, allowing integrating different tools and software, making it easier to develop complex workflows that can handle a wide range of data types. CWL also provides a flexible and expressive syntax for defining workflows. This allows users to define their workflows in a variety of programming languages, including YAML, JSON, and Python, making it easy for users with different backgrounds to adopt and use the platform. Notably, CWL workflows are designed to be reproducible, ensuring that the results obtained from a workflow are consistent across different computing environments, making it easier to reproduce and validate results across third-parties. There is a large and active community that provides support and development for CWL and the standard itself is supported by workflow frameworks such as Toil (Vivian et al., 2017). This ensures that CWL constantly improves and evolves, with new features and functionalities being added regularly. Taking a step further in resolving potential issues regarding software dependencies and compatibility, CWL also supports the execution of operations using Docker containers (Merkel, 2014). Docker is a containerization platform that allows for packaging an application along with its dependencies and running it in a self-contained unit called a container. Docker containers can run on any machine that has Docker installed, regardless of the underlying operating system or hardware. This makes it easy to move applications between environments, from development to production or from one cloud provider to another.

Here, we established automated software pipelines for analyzing RNA-Seq, ChIP-Seq and Germline variant calling experiments using CWL v1.0. Through the analysis of publicly available Illumina short-read data, we recapitulate major findings of previous studies on Chronic Lymphocytic Leukemia (CLL) and report high performance in germline variant calling using samples from the Genome in a Bottle (GIAB) Consortium, thus validating functionality and reproducibility for each software pipeline.

CLL is a heterogeneous, slow-growing type of cancer that develops when abnormal B-lymphocytes accumulate in the bone marrow and blood. These malignant cells can interfere with the production of normal blood cells, leading to anemia, infections, and bleeding disorders. CLL is the most common subtype of leukemia in adults of the western countries, accounting for approximately 30% of all cases of leukemia (Redaelli et al., 2004). The events leading to malignant transformation are gradually becoming known, with large chromosomal rearrangements, such as trisomy 12, being the probable cause of disease initiation and further accumulating somatic mutations contributing to disease aggressiveness (Landau et al., 2015). CLL patients can be distinguished in two categories with markedly different behavior and outcome based on the somatic hypermutation (SHM) status of the clonotypic stereotyped B cell receptors (BcR). Specifically, patients with no or few SHMs in their immunoglobulin heavy variable (IGHV) genes (“unmutated” CLL; U-CLL) experience a significantly more aggressive disease than those with a significant SHM load (“mutated” CLL; M-CLL) (Damle et al., 1999; Hamblin et al., 1999; Papakonstantinou et al., 2019).

The GIAB project is a collaborative effort led by the National Institute of Standards and Technology (NIST) to develop reference materials and methods for accurately measuring human genome sequences. The project aims to provide a set of well-characterized genomic reference materials that can be used to evaluate the performance of genomic sequencing technologies and inform the development of new methods for genomic analysis (Zook et al., 2014; Zook et al., 2016). The usefulness of the GIAB project lies in its ability to improve the accuracy and reliability of genomic sequencing, which is essential for applications such as personalized medicine, disease diagnosis, and drug development. Open access to standardized reference materials and methods allows for researchers to better compare and validate different sequencing technologies, which in turn improves the quality and consistency of genomic data. This can lead to more accurate diagnoses, more effective treatments, and a deeper understanding of the genetic basis of disease.

The CWL Command Line Tools and Workflows designed here were rigorously tested and validated for their performance and functionality on publicly available CLL and GIAB HTS data. All CWL-based software tools use Docker containers to automate software installation and confer cross-platform portability, and can be adopted for applications across various fields in biological sciences.

## Results

### HTS data analyses for CLL cases

#### CWL workflow for RNA-Seq

The pipeline for the processing of RNA-Seq data integrates several tools into a complete CWL workflow (Supplementary Figure S1), which receives as input raw FASTQ files from RNA-Seq experiments. The initial quality control of Illumina reads is performed using FastQC (Andrews et al., 2012) and trimming of the reads (e.g., removal of adapter and/or low quality sequences) is done using Trim galore (Krueger et al., 2021). An optional step for custom processing of the reads using FASTA/Q Trimmer (part of the FASTX-toolkit) (Hannon, 2010) is available at this point. The pre-processed reads are then mapped to the reference genome using HISAT2 (Kim et al., 2019) and samtools software (Li et al., 2009; Danecek et al., 2021) is used to convert mapped reads from SAM (Sequence Alignment Map) to BAM (Binary Alignment Map) format, and sort mapped reads based on chromosomal coordinates. Subsequently, two independent workflows are implemented for differential expression analysis at the transcript and gene level. First, following the reference protocol for HISAT, StringTie and Ballgown transcript expression analysis (Pertea et al., 2016), the program StringTie along with a reference transcript annotation GTF (Gene Transfer Format) file (if one is available) is used to:

I. Assemble transcripts for each RNA-Seq sample using the previous read alignments (BAM files)
II. Generate a global, non-redundant set of transcripts observed in any of the RNA-Seq samples
III. Estimate transcript abundances and generate read coverage tables for each RNA-Seq sample, based on the global, merged set of transcripts (rather than the reference) which is observed across all samples

The Ballgown program (Frazee et al., 2015) is then used to load the coverage tables generated in the previous step and perform statistical analyses for differential expression at the transcript level. Notably, the StringTie - Ballgown protocol applied here was selected to include potentially novel transcripts in the analysis. Second, the featureCounts software (Liao et al., 2014) is used to count reads that are mapped to selected genomic features, in this case genes by default, and generate a table of read counts per gene and sample. This table is passed as input to the DESeq2 tool (Love et al., 2014) to perform differential expression analysis at the gene level. Ballgown and DESeq2 R scripts, along with their respective CWL wrappers, were designed to receive various parameters as input, such as experimental design, contrasts of interest, numeric thresholds, and hidden batch effects.

#### Differential gene expression analysis between CLL stereotyped subsets 6 and 8

To validate the effectiveness of the CWL RNA-Seq workflow, we attempted to reproduce the analysis by Papakonstantinou et al. (Papakonstantinou et al., 2019). Specifically, we re-analyzed RNA-Seq data (150 bp long, paired-end reads), produced by the NextSeq 500 Illumina platform, for two well-characterized clinically aggressive CLL subgroups, namely, stereotyped subsets #6 (IGHV1-69/IGKV3-20) (n = 5) and #8 (IGHV4-39/IGKV1(D)-39) (n = 4) (Papakonstantinou et al., 2019). These subgroups are characterized by the presence of similar or “stereotyped” BcRs, indicating that the cancer cells have originated from a common precursor cell (clonal populations). Both stereotyped subsets #6 and #8 belong to the U-CLL category of CLL patients and present similarities beyond their SHM status. However, subset #6 has been reported to follow a more indolent course (Forconi et al., 2010) than subset #8, with the latter being more aggressive and exhibiting the highest risk of Richter transformation among all CLL cases (Rossi et al., 2009). The RNA-Seq data that were re-analyzed with our workflow concern total RNA from negatively selected CD19^+^ B-cells or peripheral blood mononuclear cells (PBMCs), which were isolated from CLL patients.

**FIGURE 1:**
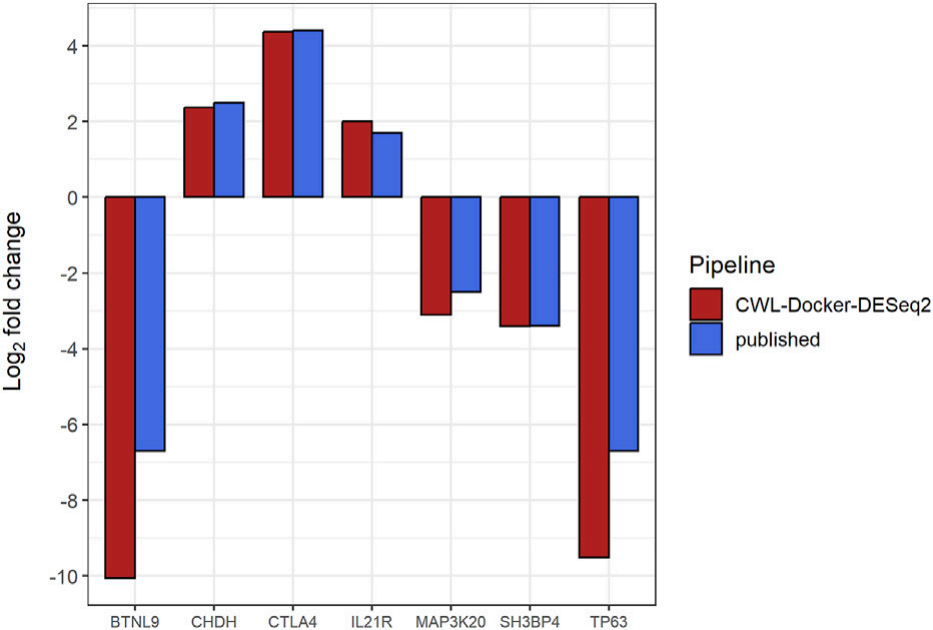
Bar plot showing the Log2 fold changes (*y*-axis) in expression levels for seven genes (*BTNL9*, *CHDH*, *CTLA4*, *IL21R*, *SH3BP4*, *ZAK*, *TP63*) (*x*-axis) that were detected to be differentially methylated and expressed in the original study (genes of interest), following comparisons between CLL stereotyped subsets #6 and #8 [#6 (numerator) vs. #8 (denominator)] using the CWL-based RNA-Seq workflow. Both published and CWL-based RNA-Seq workflow results follow the same pattern of expression differences for all genes of interest (Papakonstantinou et al., 2019).

**FIGURE 2:**
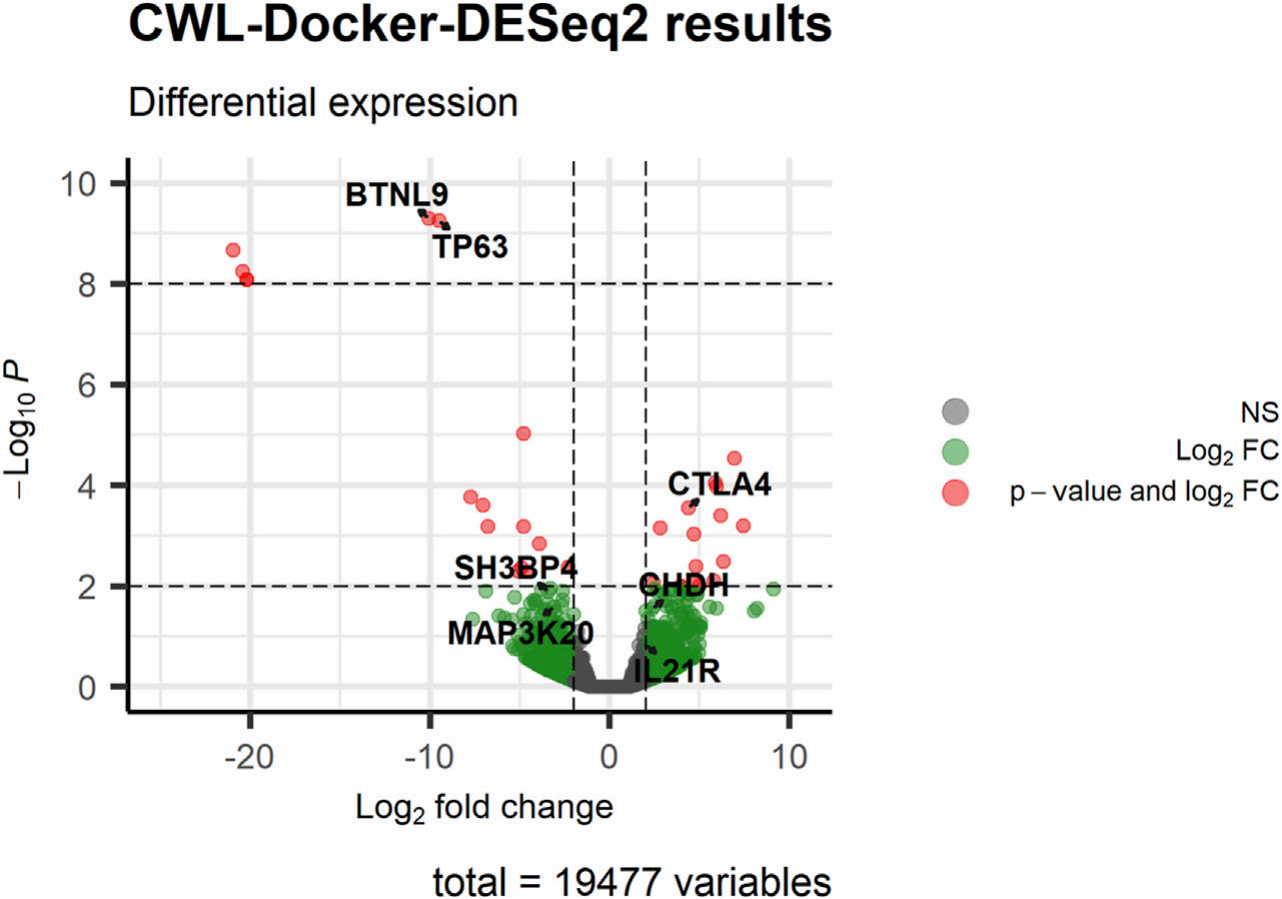
Visualizing the results of the differential gene expression analysis performed by CWL-based RNA-Seq pipeline using the RNA-Seq data of CLL stereotyped subsets #6 and #8 [#6 (numerator) vs. #8 (denominator)]. The volcano plot displays the results of the differential gene expression analysis for all genes. Genes of interest, that were detected to be differentially methylated and expressed in the original study, are highlighted to display the statistically significant levels of differential expression. Among them is *TP63*, which was reported to act as a pro-survival biomarker for CLL subset #8 cells (Papakonstantinou et al., 2019).

Following pre-processing and differential gene expression analysis with the RNA-Seq workflow, we detected 99 differentially expressed genes (log2FC ≥ 1, *p*-value ≤0.05), 38 of which were common to the 321 differentially expressed genes reported by the original study using the same filtering criteria (Supplementary Figure S2). Notably, we successfully detected as differentially expressed, between subsets #6 and #8, the 7 genes (namely, *BTNL9*, *CHDH*, *CTLA4*, *IL21R*, *SH3BP4*, *ZAK*, and *TP63*), with the exception of *IL21R* having log2FC ≥ 1 (∼2) but *p*-value ∼0.15, that were detected to be differentially methylated and expressed in the original study (Figure 1; Figure 2). It is of note that we successfully detected the differentially expressed *TP63* (FDR-adjusted *p*-value <0.001), as this gene was reported to be hypomethylated and overexpressed in subset #8, and its encoded protein (p63) was shown to act as a pro-survival factor in CLL models *in vitro* (Papakonstantinou et al., 2019).

#### CWL workflow for ChIP-Seq

Similarly to the CWL-based RNA-Seq pipeline, the pipeline for processing of ChIP-Seq data integrates various tools and receives raw FASTQ files as input (Supplementary Figure S3). The initial quality control of Illumina reads is performed using FastQC, while the trimming processes are executed using Trimmomatic (Bolger et al., 2014). The pre-processed reads are then mapped to the reference genome using HISAT2 and samtools is used to convert mapped reads from SAM to BAM format, sort mapped reads based on read names, add information regarding paired-end reads (e.g., CIGAR field information), resort based on chromosomal coordinates, remove duplicate reads and create indexes on coordinate-sorted BAM files for fast random access. At this point, quality metrics and files for inspecting the mapped ChIP-Seq reads, considering the experimental design, are produced independently using deeptools2 (Ramírez et al., 2016). Briefly:

I. Read coverages for genomic regions of two or more BAM files are computed (multiBamSummary). The results are produced in compressed numpy array (NPZ) format and are used to calculate and visualize pairwise correlation values between the read coverages (plotCorrelation).
II. Estimation of sequencing depth, through genomic position (base pair) sampling, and visualization is performed for multiple BAM files (plotCoverage).
III. Cumulative read coverages for each indexed BAM file are plotted by counting and sorting all reads overlapping a “window” of specified length (plotFingerprint).
IV. Production of coverage track files (bigWig), with the coverage calculated as the number of reads per consecutive windows of predefined size (bamCoverage), and normalized through various available methods (e.g., Reads Per Kilobase per Million mapped reads; RPKM). The coverage track files are used to calculate scores per selected genomic regions (computeMatrix), typically genes, and a heatmap is produced based on the scores associated with these genomic regions (plotHeatmap).

Subsequently, the process of calling potential binding positions (peaks) to the genome (peak calling) is executed using MACS2 (Zhang et al., 2008), which produces a series of BED (Browser Extensible Data) files in different formats.

Central part of the CWL ChIP-Seq workflow is the differential binding analysis, which aims to detect epigenetic changes, such as altered levels of transcription factor binding or histone modifications, that are connected to different experimental conditions (phenotypic traits, different drug treatments, etc.). For this purpose, quality control of ChIP-Seq data is performed with ChIPQC (Carroll et al., 2014) and differential binding analysis is executed, using by default the narrowPeak (BED) files produced by MACS2, with DiffBind (Ross-Innes et al., 2012). Furthermore, the ROSE (Rank Ordering of Super-Enhancers) software was integrated into the workflow for detecting regions which act as super-enhancers (Lovén et al., 2013; Whyte et al., 2013). Super-enhancers are defined as genomic regions with high concentration of enhancer elements that are considered to be functionally clustered and act synergistically to regulate the transcription of genes involved in critical functions such as cell differentiation (Lovén et al., 2013; Whyte et al., 2013). The detection of super-enhancers by ROSE is followed by repetition of the ChIPQC and DiffBind analyses solely for these regions. In addition, for the application of custom analyses on MACS2 peak calling results, a table containing the total number of reads per peak (rows) and for all samples (columns), is generated by utilizing bedtools (Quinlan and Hall, 2010) software and integrating a series of independent steps in the workflow. It is worth noting that wherever is necessary, the option of removing peaks overlapping with known, problematic regions of the genome (i.e., blacklisted regions) (Amemiya et al., 2019) is provided to avoid miscalculations, such as overestimation of peak intensity, and wrong interpretation of the results.

#### Differential binding analysis between CLL subgroups with mutated and unmutated IGHV gene status

The CWL ChIP-Seq workflow was tested by re-analyzing ChIP-Seq data (50 bp long, paired-end reads), produced by the HiSeq 2000 Illumina platform, for different subgroups of CLL patients that are distinguished by the presence (M-CLL) or absence (U-CLL) of IGHV gene mutations. Specifically, we attempted to reproduce the analysis of Kosalai et al. (Kosalai et al., 2019) on samples that originate from the peripheral blood of M- and U-CLL patients and were subjected to immunoprecipitation for EZH2 methyltransferase and H3K27me3 histone modification, to identify sites where EZH2 binds and catalyzes the H3K27me3 modification with different efficiency between the aggressive U-CLL (n = 6) and the more indolent M-CLL (n = 6) (Kosalai et al., 2019).

**FIGURE 3:**
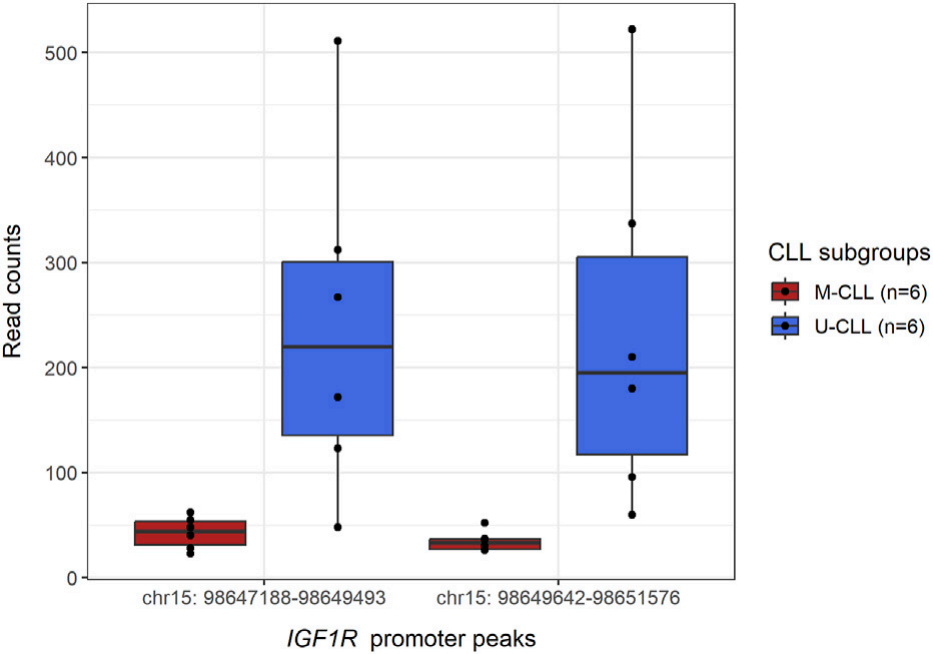
Boxplot displaying the differential binding of EZH2 methyltransferase in the region of *IGF1R* promoter, between M- and U-CLL subgroups. In particular, DiffBind analysis of the U-CLL samples detected two peaks (chr15:98650048-98650448 and chr15:98648126-98648526) in which EZH2 is bound in a statistically significant manner (FDR-adjusted *p*-value <0.001) in U-CLL samples compared to the respective regions in M-CLL samples (see Supporting data). From the table containing the consensus peak regions and their respective read counts, the read counts (*y*-axis) for the consensus peak regions (*x*-axis) overlapping with the DiffBind-detected *IGF1R* promoter peaks were extracted and shown here.

The CWL ChIP-Seq workflow proved effective in detecting the statistically significant, differential binding of EZH2 to the promoter region of the *IGF1R* gene in U-CLL compared to M-CLL (FDR-adjusted *p*-value <0.001 for peaks chr15:98650048-98650448 and chr15:98648126-98648526) (Figure 3 and Supplementary Figure S4), while at the same time the levels of H3K27me3 remained unchanged between the two subgroups for the same region (Supplementary Figure S5). These results reflect the main findings of the published study, where it was shown that *IGF1R* is non-canonically activated in U-CLL through the EZH2-mediated recruitment of MYC in its promoter, resulting in further activation of the PI3K pathway (Kosalai et al., 2019).

### Germline variant calling for GIAB samples

#### CWL workflow for germline variant calling

We developed a complete pipeline for calling small germline variants, namely, SNPs and small INDELs, by processing data from whole- (WGS) or targeted-genome sequencing experiments (e.g., whole-exome sequencing; WES). The pipeline receives as input FASTQ files and includes some pre-processing steps. As with the RNA-Seq workflow, the initial quality control and trimming processes are performed using FastQC and Trim galore, respectively. The pre-processed reads are then mapped to the reference genome using the bwa-mem algorithm (Li, 2013). Samtools software is then applied to convert mapped reads from SAM to BAM format, sort mapped reads based on read names, add information regarding paired-end reads (e.g., CIGAR field information), and resort based on chromosomal coordinates. Subsequently, Picard Tools software (Picard Tools, 2021) is used to: i) add basic Read-Group information regarding sample name, platform unit, platform (e.g., ILLUMINA), library and identifier (picard AddOrReplaceReadGroups), and ii) mark PCR and/or optical duplicate reads (picard MarkDuplicates), to each BAM file. Samtools software is additionally used to collect summary statistics and create indexes on coordinate-sorted BAM files for fast random access. At this point, following the recommendations of Genome Analysis Toolkit (GATK) for best practices in germline variant calling (DePristo et al., 2011; Van der Auwera and O’Connor, 2020), the pipeline is split into two separate workflows to process samples differently during the variant detection and filtering steps. For both workflows, to reduce time and take advantage of all available computational resources, the reference genome is split into a predefined number of intervals, using the GATK SplitIntervals tool (Van der Auwera et al., 2013), for parallel processing through incorporation of CWL subworkflows and application of the “scatter and gather” approach. The developed workflows include:

I. Multi-sample workflow (Supplementary Figure S6): Following the variant calling step, multiple samples are concatenated into a single, unified VCF (Variant Calling Format) file, which contains the variant information for all samples. In particular, using the GATK BaseRecalibrator and ApplyBQSR tools (McKenna et al., 2010), BAM files are firstly evaluated with a process called Base Quality Score Recalibration (BQSR), during which Machine Learning (ML) models are used to detect miscalculated base quality scores, due to potential sources of non-random technical errors, and re-estimate them. Variant calling is then performed using GATK HaplotypeCaller (Poplin et al., 2018) in gVCF (genomic VCF) mode (-ERC GVCF) and all genomic interval-split gVCF files of each sample are merged using the GATK MergeVCFs tool. The unified VCF file is then produced using the GATK CombineGVCFs and GenotypeGVCFs tools, and it is further annotated, separately for SNP and INDEL variants, using the Variant Quality Score Recalibration (VQSR) method (DePristo et al., 2011). VQSR employs ML algorithms to create models of true and false variant calls and utilize them to estimate new quality scores for the detected variants. It is important to note here that both BQSR and VQSR require dataset(s) of known and well-characterized variants (e.g., from dbSNP or 1,000 Genomes for *Homo sapiens*) to train their respective ML models and apply score recalculation accurately. In the final steps of the analysis, the bcftools software (Li et al., 2009; Danecek et al., 2021) is used to filter the variants, based on the information added during VQSR and/or custom filters, and normalize INDELs (split multiallelic sites). The ANNOVAR tool (Wang et al., 2010) is then used to annotate the final dataset of filtered variants with genomic, population-related and/or clinical information.
II. Single-sample workflow (Supplementary Figure S7): Accepting as input multiple samples, which are not merged into a unified VCF file but are rather processed separately in each step of the workflow, leading to the production of a VCF file for each sample. In this approach, BQSR and variant calling with HaplotypeCaller also take place as the initial steps. Next, the GATK CNNScoreVariants is applied, a tool that streams variants and their reference context to a python program, which evaluates a pre-trained Convolutional Neural Network (CNN) on each variant and annotates each VCF with variant call scores (DePristo et al., 2011; Van der Auwera and O’Connor, 2020). VCF files are further annotated (tranche filtering) using the CNN-annotated variant scores of the INFO field and the GATK FilterVariantTranches tool. Finally, filtering and INDEL normalization with bcftools and annotation with ANNOVAR are performed for each VCF. The workflow also incorporates an independent, optional step of variant hard filtering, where hard filters, based on GATK4 suggestions, are applied to each VCF file instead of the CNN pre-trained model-based filtering.

It is worth noting that a different combination of additional GATK tools and CWL subworkflows is used to apply BQSR in each workflow. Additionally, variations of the described workflows, omitting BQSR, VQSR and CNN pre-trained model-based filtering and using only hard filtering are currently under development (“dev” branch of GitHub repository). These alternative workflows will constitute simple but ready-to-use solutions for the analysis of samples from non-reference organisms lacking the required, well-defined variant datasets for ML-based variant filtration.

### Comparison of germline variant calling concordance

The CWL Germline Variant calling workflow was validated for its functionality and accuracy using raw data from the GIAB project (Zook et al., 2014; Zook et al., 2016). In particular, we utilized WGS data of the reference GIAB samples (golden call sets) of: i) NA12878, that refers to the genome of a B-lymphocytic cell line, and ii) NA24385, NA24149 and NA24143, that correspond to the son, father and mother of an Ashkenazi family trio (see Methods). These samples were originally sequenced and processed by NIST, using multiple sequencing platforms and computational tools for mapping and variant calling, for the purpose of creating reliable genomic data resources that can be used to assess the performance of novel germline variant calling software tools (Krusche et al., 2019). Thus, the raw WGS data of GIAB samples are accompanied by reference VCF files (truth sets) which contain the majority of confirmed SNP and small INDEL variants for each sample (∼80% genome coverage).

**FIGURE 4:**
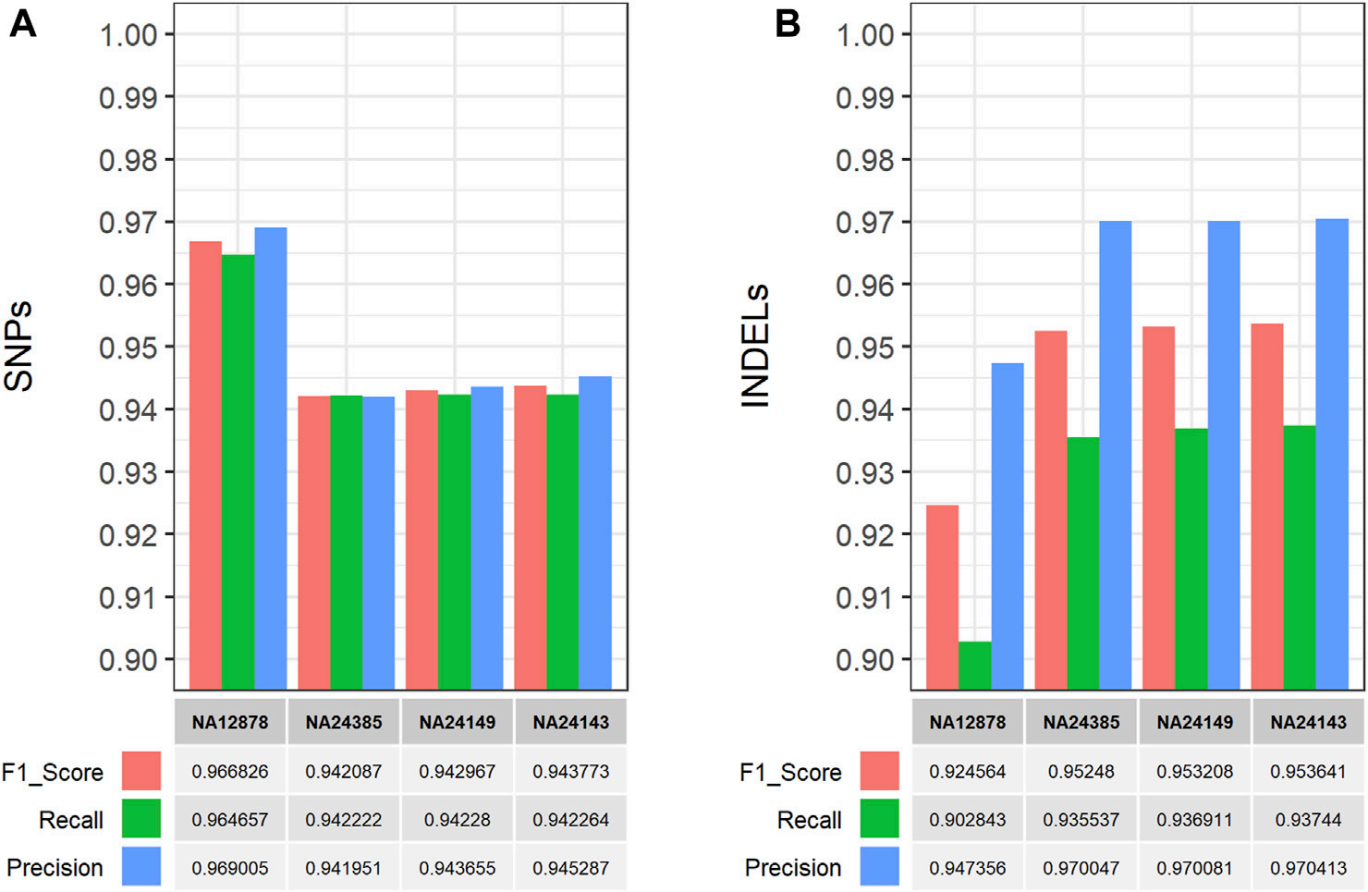
Performance evaluation of the CWL-based (single-sample) germline variant calling pipeline on Illumina short-read, WGS data from four GIAB samples (Zook et al., 2014; 2016). The CWL workflow was applied on all GIAB samples and performance metrics were calculated using hap. py (https://github.com/Illumina/hap.py) for comparisons between the detected and filtered **(A)** SNPs and **(B)** INDELs, against the respective gold standard VCF truth set of each sample. The workflow performed well on all samples, achieving >90% for all performance metrics in both SNP and INDEL comparisons.

**FIGURE 5:**
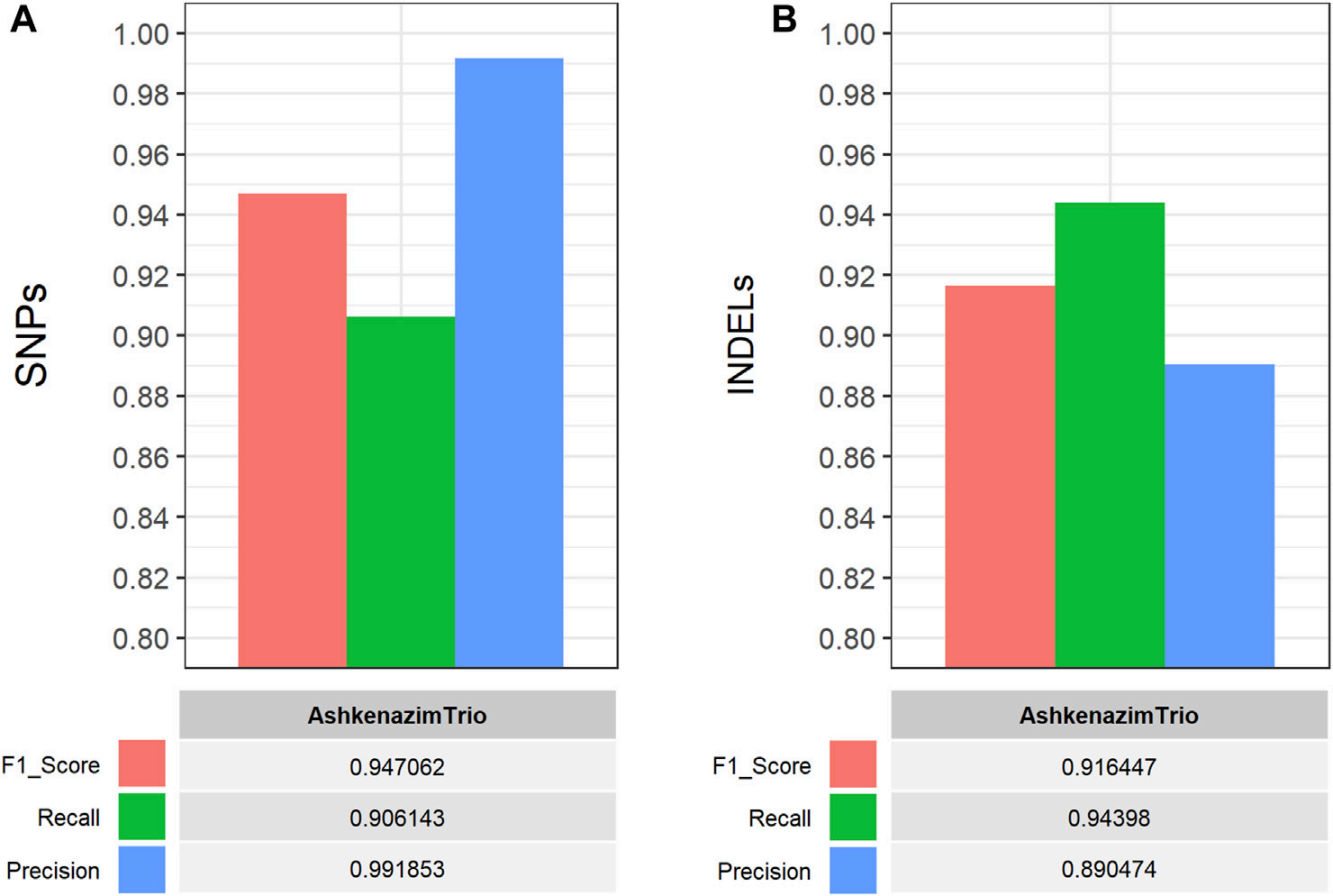
Performance evaluation of the CWL-based (multi-sample) germline variant calling pipeline on Illumina short-read, WGS data from three GIAB samples (AshkenazimTrio) (Zook et al., 2014; 2016). The CWL workflow was applied on all GIAB samples and performance metrics were calculated using hap. py (https://github.com/Illumina/hap.py) for comparisons between the detected and filtered **(A)** SNPs and **(B)** INDELs, against the respective gold standard VCF truth set of all samples. The latter was produced by merging all gold standard truth set VCF files for the Ashkenazi family trio samples into a single VCF file for comparison, using bcftools. All performance metrics exceeded 90%, with the exception of Precision in INDEL comparisons (∼89%), validating the functionality of the multi-sample workflow.

The performance of the single-sample germline variant calling workflow was evaluated on all four GIAB samples by performing comparisons between the detected short variants, which were filtered based on CNN-annotated variant scores, and the respective gold standard VCF truth sets. The hap. py software (https://github.com/Illumina/hap.py) was used to perform genotype-level, haplotype comparisons and calculate the performance metrics (F1 score, Recall, Precision). Using default parameters, the workflow performed well on all GIAB samples with >90% for all performance metrics in both SNP and INDEL comparisons (Figure 4), thus validating its functionality and applicability. Similarly, the multi-sample germline variant calling workflow was evaluated by comparing the detected variants of the Ashkenazi family trio samples. Specifically, all gold standard truth set VCF files for the Ashkenazi family trio samples were merged using bcftools into a single VCF file for comparison with the detected and filtered variants of the workflow, which are also reported in a single VCF file. Similarly, the high confidence genomic regions (BED format) for each sample, that are used for benchmarking purposes, were concatenated, sorted and merged using bedtools, to be used with hap. py for the variant comparison. As with the single-sample workflow, all performance metrics exceeded 90%, with the exception of precision in INDEL comparisons (∼89%), validating the functionality of the multi-sample workflow (Figure 5).

## Discussion

Rapid technological advancements, development of new methodologies and software tools as well as the ever-increasing volume of available multi-omics data, have increased the complexity of *in silico* analyses and placed a strain in reproducibility and reusability efforts. The high number of non-reproducible experimental results, retracted articles and failing clinical trials calls for the establishment of a minimum set of standards required to back scientific claims (Sandve et al., 2013), with several systematic efforts being undertaken towards this goal, such as FAIR Principles (Wilkinson et al., 2016; Goble et al., 2020; Barker et al., 2022) and DOME recommendations (Walsh et al., 2021). Having the ability to accurately reproduce scientific results leads to increased productivity, due to being able to easily reuse and/or repurpose previous methods and code in new projects, and makes one’s research more accessible and trustworthy as well. In this regard, the utilization of platforms like Galaxy (Afgan et al., 2018), and the adoption of workflow frameworks [Nextflow (Di Tommaso et al., 2017), Snakemake (Köster and Rahmann, 2012)] and specifications [CWL (Chapman et al., 2016), RO-Crate (Soiland-Reyes et al., 2022), Biocompute Object (Vahan Simonyan et al., 2017)] offers several advantages in the development of reproducible software pipelines.

CWL constitutes an open standard for defining computational workflows in a platform-agnostic manner, facilitating significant workflow automation, reproducibility and reusability. Its increasing popularity has led to the design of sophisticated software solutions, including novel, open-source pipelines (Korhonen et al., 2019) and dedicated workflow managers (Kotliar et al., 2019), based on this open standard, while several popular workflow managers such as Toil or the commercial Seven Bridges platform, are adapting to use it. For the CWL workflows designed here, we opted to utilize publicly available and custom-made Docker images (“pull actions”) to run separate containers for all integrated software tools. Docker offers scalability, easy deployment and provides a consistent, isolated runtime environment for applications, regardless of the host operating system or infrastructure. This makes it easier to maintain and update applications, and reduces the likelihood of compatibility issues.

However, there are some security concerns regarding the use of Docker. Specifically, Docker containers are designed to run with process-level isolation, providing a level of security by isolating applications and their dependencies. However, Docker containers typically run with root-level permissions by default, which introduces potential security risks (T. Combe et al., 2016). Best practices, such as running containers with limited privileges and employing additional security measures, should be followed to enhance the security of Docker deployments. Adoption of CWL-supported software solutions including Singularity (Kurtzer et al., 2017), which was designed for high-performance computing (HPC) environments and places a strong emphasis on security and isolation, or udocker (Gomes et al., 2018), an alternative Docker implementation allowing for execution of Linux containers in user mode, can also help in mitigating security risks. Furthermore, it is worth mentioning that sometimes common processes taking place between workflow steps, including file(s) manipulation or execution of conditional statements, requires the application of workarounds such as implementation of JavaScript-based “expression tools”. Although this may burden the workflow with increased verbosity, it nevertheless attests to the flexibility offered by CWL.

Using previously published data, we established the functionality of three CWL pipelines designed for processing HTS data originating from RNA-Seq, ChIP-Seq and germline variant calling experiments. Namely, through the application of: i) the RNA-Seq workflow we confirmed the overexpression of pro-survival *TP63* in subset #8 CLL patients compared to #6 subset (Papakonstantinou et al., 2019), and ii) the ChIP-Seq workflow we confirmed the non-canonical, differential binding of EZH2 to the promoter of *IGF1R*, whose expression is non-canonically induced in U-CLL patients by EZH2-recruited MYC leading to the activation of the PI3K pathway (Kosalai et al., 2019). Moreover, we displayed the high performance of the germline variant calling pipeline through processing of publicly available WGS data from the GIAB project and comparison with the respective VCF truth set data (Zook et al., 2014, Zook et al.,2016). These findings support the use or adaptation of the CWL pipelines developed here as reliable and reproducible software solutions to routinely performed HTS experiments aiming for biomarker discovery. Furthermore, it is our hope that these workflows will constitute an open resource that will facilitate the adoption of CWL in future software pipeline development.

## Methods

### Reference data acquisition

The RNA-Seq data from CLL stereotyped subsets #6 and #8 are available from the ArrayExpress database at EMBL-EBI (www.ebi.ac.uk/arrayexpress), under the accession number E-MTAB-6962. Additional information on CLL RNA-Seq data (accession numbers, sample names, etc.) are available in Supplementary Table S1. The EZH2 and H3K27me3 ChIP-Seq data for M-CLL and U-CLL subgroups are available from the Gene Expression Omnibus (GEO) NCBI database (https://www.ncbi.nlm.nih.gov/geo/), under the accession number GSE115772. The publicly available WGS data used for the four GIAB samples, are available from SRA (https://www.ncbi.nlm.nih.gov/sra), under the accession numbers SRR6794144 (NA12878, B-lymphocytic cell line), SRR22476789 (AshkenazimTrio son), SRR22476790 (AshkenazimTrio father), and SRR22476791 (AshkenazimTrio mother).

### CWL pipelines

The pipelines developed here follow the syntax specified in CWL v1.0 (Chapman et al., 2016). A CWL wrapper was written for each software tool using CWL CommandLineTool syntax, and the tools were integrated into functional workflows using CWL Workflow syntax. The workflows were operated using the cwltool package v3.1.20221018083734 (https://github.com/common-workflow-language/cwltool). A number of CWL wrappers (Command Line Tools), that were utilized in our workflows, were adapted from publicly available sources. We provide a list of these tools and their respective sources in Supplementary Table S2. For the execution of each tool publicly available Docker images from Docker Hub and Quay. io were used, with the exception of Ballgown, DESeq2, ChIPQC, DiffBind and ROSE, for which custom scripts were written and incorporated into in-house Docker images. These in-house Docker images are available in Docker Hub (https://hub.docker.com/u/biodataanalysisgroup) and their respective Dockerfiles can be found in the CWL pipelines GitHub repository (https://github.com/BiodataAnalysisGroup/CWL_HTS_pipelines). A list of all software tools and their respective Docker images is available in Supplementary Table S3. Docker v20.10.19 was used to pull and create Docker containers. A list of dependency files (e.g., reference genome files) for the execution of each workflow is also available in Supplementary Table S4. The workflows are ordered in a way by which the output data of each step constitute the required input data of one or more of the next steps. Wherever possible the “scatter” feature of CWL was applied to facilitate parallel execution. The desired output files, from any of the workflow steps, can be selected from the “outputs” section of each workflow. All workflows were registered and are also available in WorkflowHub (https://workflowhub.eu/) (Goble et al., 2021).

### Additional CWL tools and workflows

Additional, short scale CWL workflows were designed for pre-processing of raw FASTQ files before executing one of the main CWL workflows. These include:

I. FastQC and MultiQC (Ewels et al., 2016) workflows for quality inspection of multiple FASTQ files
II. Trim galore and Trimmomatic workflows for sequence trimming of FASTQ files prior to the execution of the main workflow

This allows users to inspect the input data before proceeding with the main analysis. Quality control and trimming steps are optionally available. Thus, these operations can be applied independently beforehand and their output can be provided as input to the next steps of the workflow (i.e., mapping pre-processed reads).

### Pipeline runtimes

We used a 28-core Unix cluster with 238 Gb RAM for the execution of all CWL pipelines. The runtimes of all pipelines, based on the publicly available HTS data utilized for their performance evaluation, are reported at Table 1.

**TABLE 1:** Runtimes for all CWL-based pipelines, along with information on publicly available HTS data that were used for performance evaluations.

	RNA-seq	ChIP-seq (EZH2)	ChIP-seq (H3K27me3)	Single-sample germline variant calling pipeline (cwltool --parallel)	Multi-sample germline variant calling pipeline (cwltool --parallel)
Runtime	∼5 h	∼18 h	∼18 h	∼4 days and 11 h	∼2 days and 5 h
Number of (No.) samples	9	14 (with two control samples for M- and U-CLL)	14 (with two control samples for M- and U-CLL)	4	3
Average No. of reads per (paired-end) sample (million reads)	∼32.58	∼52.72	∼57.3	∼814.3	∼833.06
Read length (bp)	150	50	50	150	150

These numbers are indicative of the execution time of each pipeline when running in a single machine for a standard HTS analysis, and can be further scaled down upon configuration and parallel execution in multi-node HPC environments.

## Availability of supporting source code and requirements

Project name: Biodata Analysis Group CWL pipelines.

Project home page: https://github.com/BiodataAnalysisGroup/CWL_HTS_pipelines.

DockerHub: https://hub.docker.com/u/biodataanalysisgroup.

WorkflowHub:

• RNA-Seq: https://doi.org/10.48546/WORKFLOWHUB.WORKFLOW.524.1
• ChIP-Seq: https://doi.org/10.48546/WORKFLOWHUB.WORKFLOW.525.1
• Germline Variant calling (Multi-sample): https://doi.org/10.48546/WORKFLOWHUB.WORKFLOW.526.1
• Germline Variant calling (Single-sample): https://doi.org/10.48546/WORKFLOWHUB.WORKFLOW.527.1

Operating system(s): Linux-based systems (Ubuntu 20.04.6 LTS).

Programming language: CWL v1.0, Python, R, Bash.

Other requirements: cwltool package v3.1.20221018083734, Docker v20.10.19.

License: MIT.

Availability of supporting data: The following datasets, supporting the results of this article, are available at Zenodo repository (Kyritsis et al., 2023) and include:

• Differential transcript and gene expression results produced during the analysis with the CWL-based RNA-Seq pipeline
• Bigwig and narrowPeak files, differential binding results, table of consensus peaks and read counts of EZH2 and H3K27me3, produced during the analysis with the CWL-based ChIP-Seq pipeline
• VCF files containing the detected and filtered variants, along with the respective hap. py results regarding comparisons against the GIAB golden standard truth sets for both CWL-based germline variant calling pipelines

## Data Availability

The original contributions presented in the study are included in the article/Supplementary Material, further inquiries can be directed to the corresponding author.

## Abbreviations

BAM, Binary Alignment Map; BcR, B cell receptors; BED, Browser Extensible Data; BQSR, Base Quality Score Recalibration; ChIP, Chromatin Immunoprecipitation; CNN, Convolutional Neural Networks; CLL, Chronic Lymphocytic Leukemia; CWL, Common Workflow Language; ENA, European Nucleotide Archive; GATK, Genome Analysis Toolkit; GEO, Gene Expression Omnibus; GIAB, Genome In a Bottle; GTF, Gene Transfer Format; HTS, High-throughput Sequencing; IGHV, Immunoglobulin heavy variable genes; INDEL, Insertion—Deletion; M-CLL, Mutated CLL; NIST, National Institute of Standards and Technology; ROSE, Rank Ordering of Super-Enhancers; SAM, Sequence Alignment Map; SNP, Single-nucleotide polymorphism; SHM, Somatic Hypermutation; SRA, Sequence Read Archive; TCGA, The Cancer Genome Atlas; U-CLL, Unmutated CLL; VCF, Variant Calling Format; VQSR, Variant Quality Score Recalibration; WES, Whole-exome Sequencing; WGS, Whole-genome Sequencing.
