# Supplementary material for: Software pipelines for RNA-Seq, ChIP-Seq and germline variant calling analyses in common workflow language (CWL)
Source: Front Bioinform. 2023 Nov 7;3:1275593. doi: 10.3389/fbinf.2023.1275593 (PMC10662043; doi:10.3389/fbinf.2023.1275593)
Supplement: Supplementary file 1 [file DataSheet1.PDF]

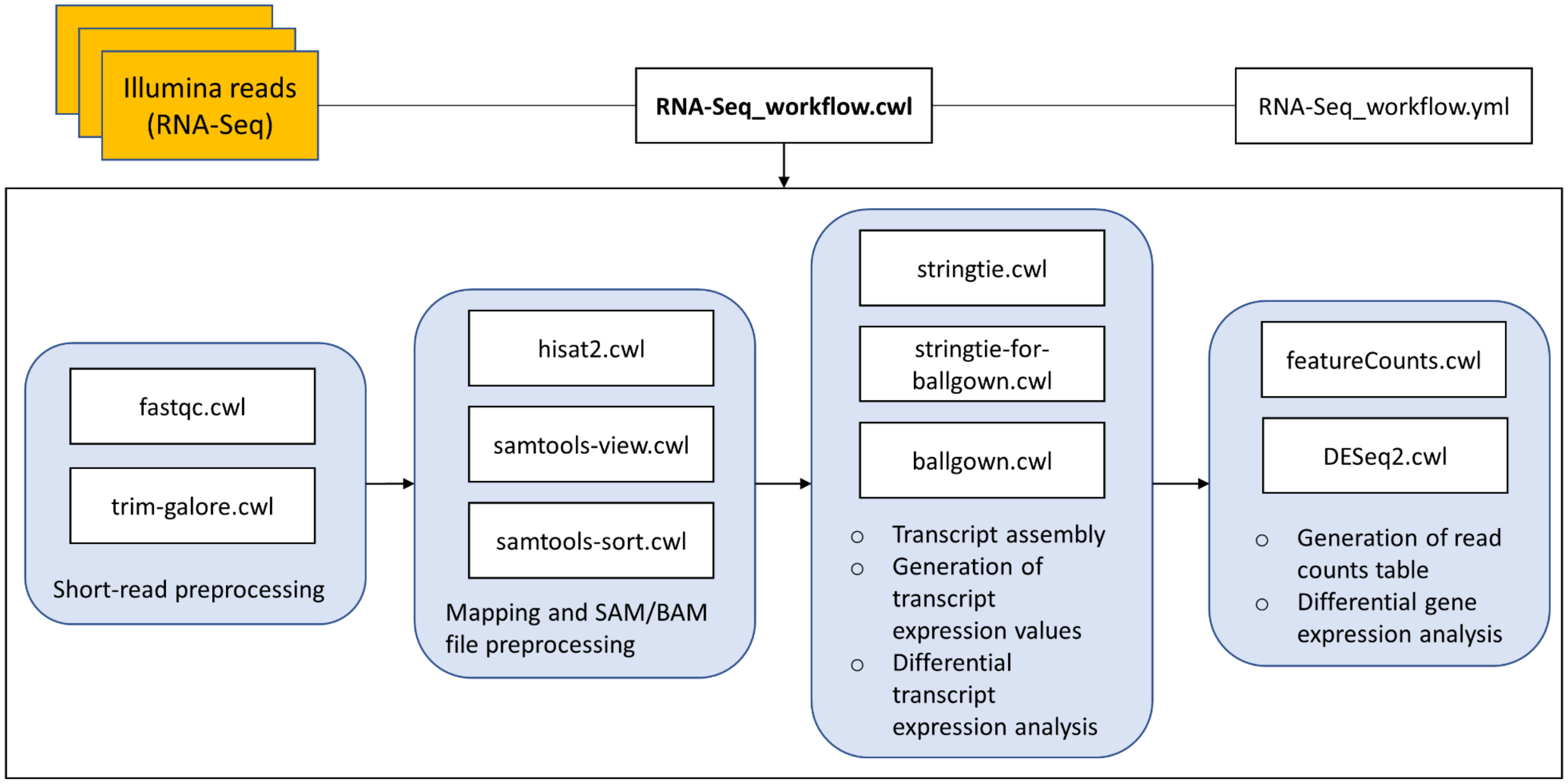

COMMON WORKFLOW LANGUAGE

docker

Figure S1: Diagram illustrating the main steps of the automated, CWL-based RNA-Seq pipeline designed for the analysis of short-read data. Briefly, the workflow integrates quality control (QC), trimming, mapping and preprocessing steps with the goal of performing differential expression for various genomic elements (transcript- and gene-level by default). The code for the workflow and the Dockerfiles for the in-house Docker images is available in the respective GitHub repository. Several steps of CWL ExpressionTools performing tasks such as moving/copying file(s) were excluded from the diagram for visualization purposes.

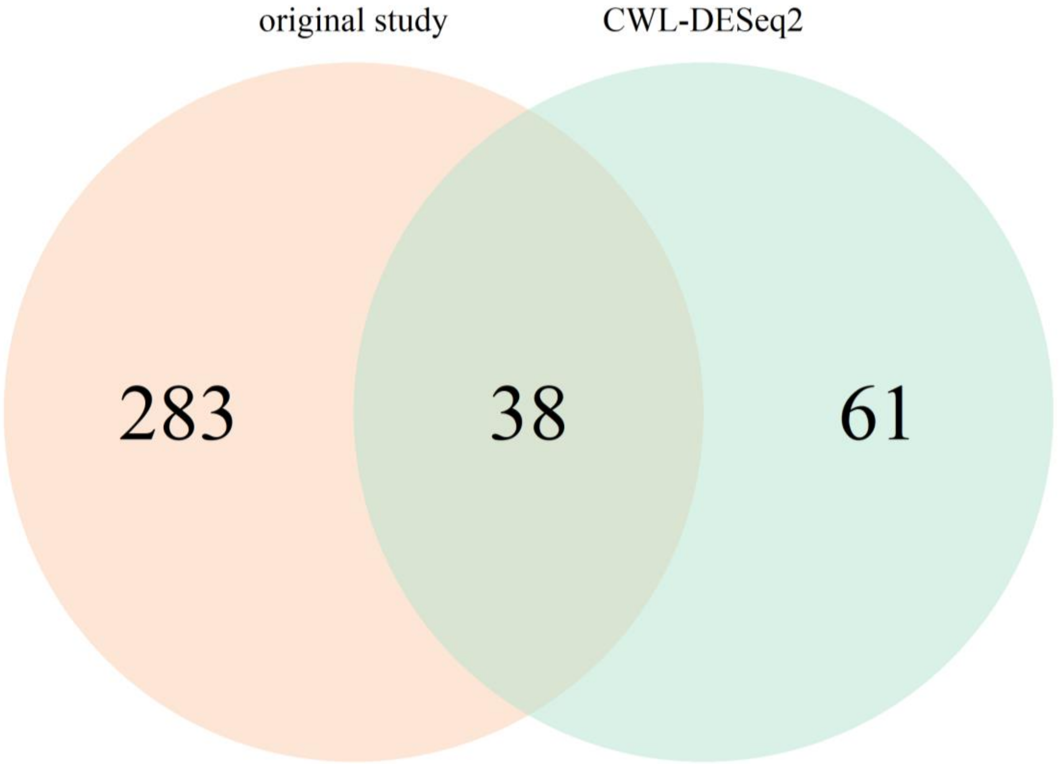

Figure S2: Venn diagram displaying the number of **common** (38) and **different genes** that were reported to be differentially expressed (**log2FC ≥ 1, p-value ≤ 0.05**) between CLL stereotyped subsets #6 and #8, in the original study [1] (left) and using the CWL-based RNA-Seq workflow (right). All files for reproducing the comparison, are available in Zenodo [2].

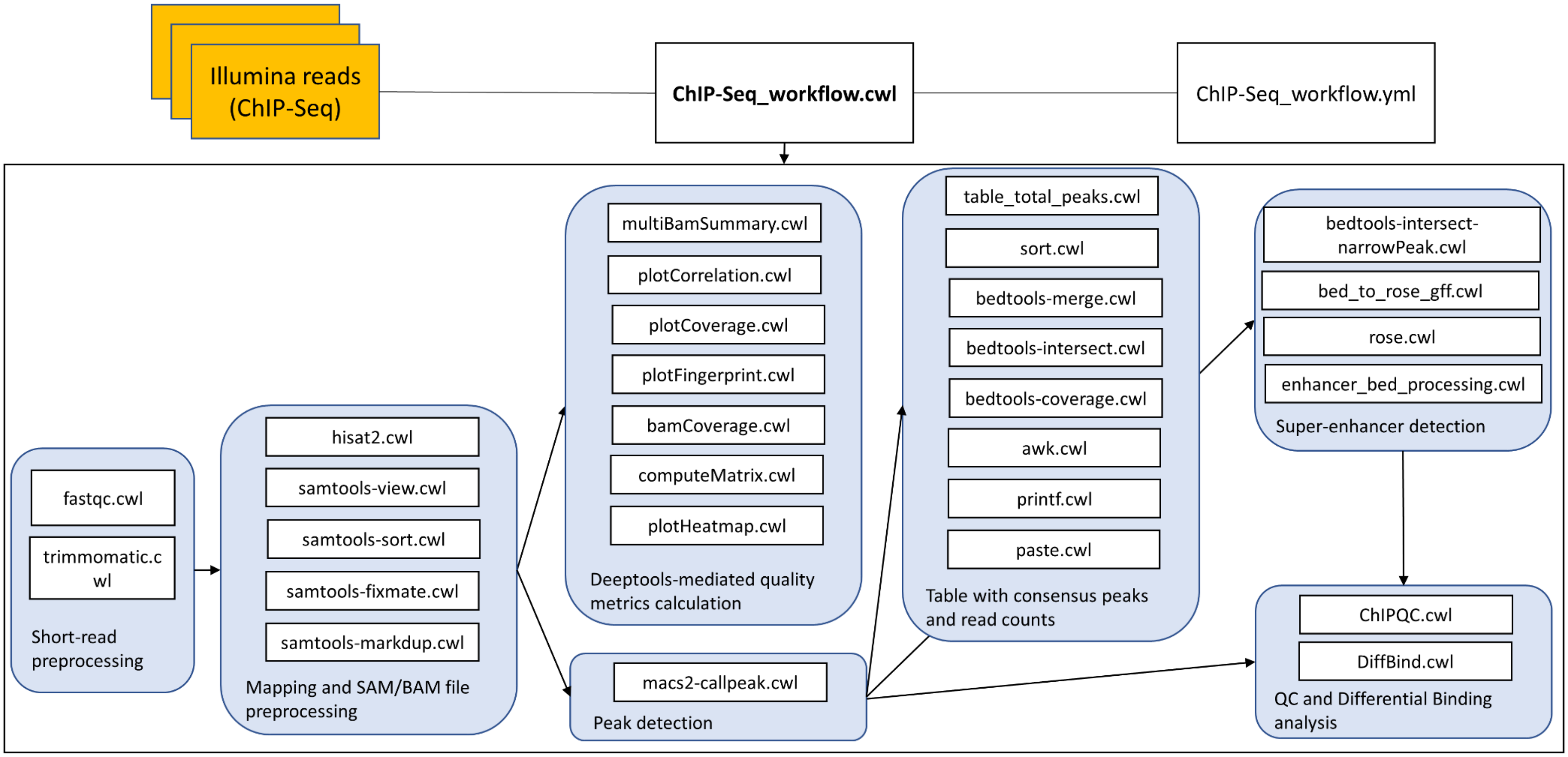

Figure S3: Diagram illustrating the main steps of the automated, CWL-based ChIP-Seq pipeline designed for the analysis of short-read data. Briefly, the workflow integrates quality control (QC), trimming, mapping and preprocessing steps with the goals of performing: (i) Peak detection using MACS2, (ii) calculation of consensus peaks and read counts, (iii) Super-enhancer detection with ROSE, and (iv) differential binding analysis for both MAC2-detected peaks and ROSE-detected super enhancers. The code for the workflow and the Dockerfiles for the in-house Docker images are available in the respective GitHub repository. Several steps of CWL ExpressionTools performing tasks such as moving/copying file(s) were excluded from the diagram for visualization purposes.

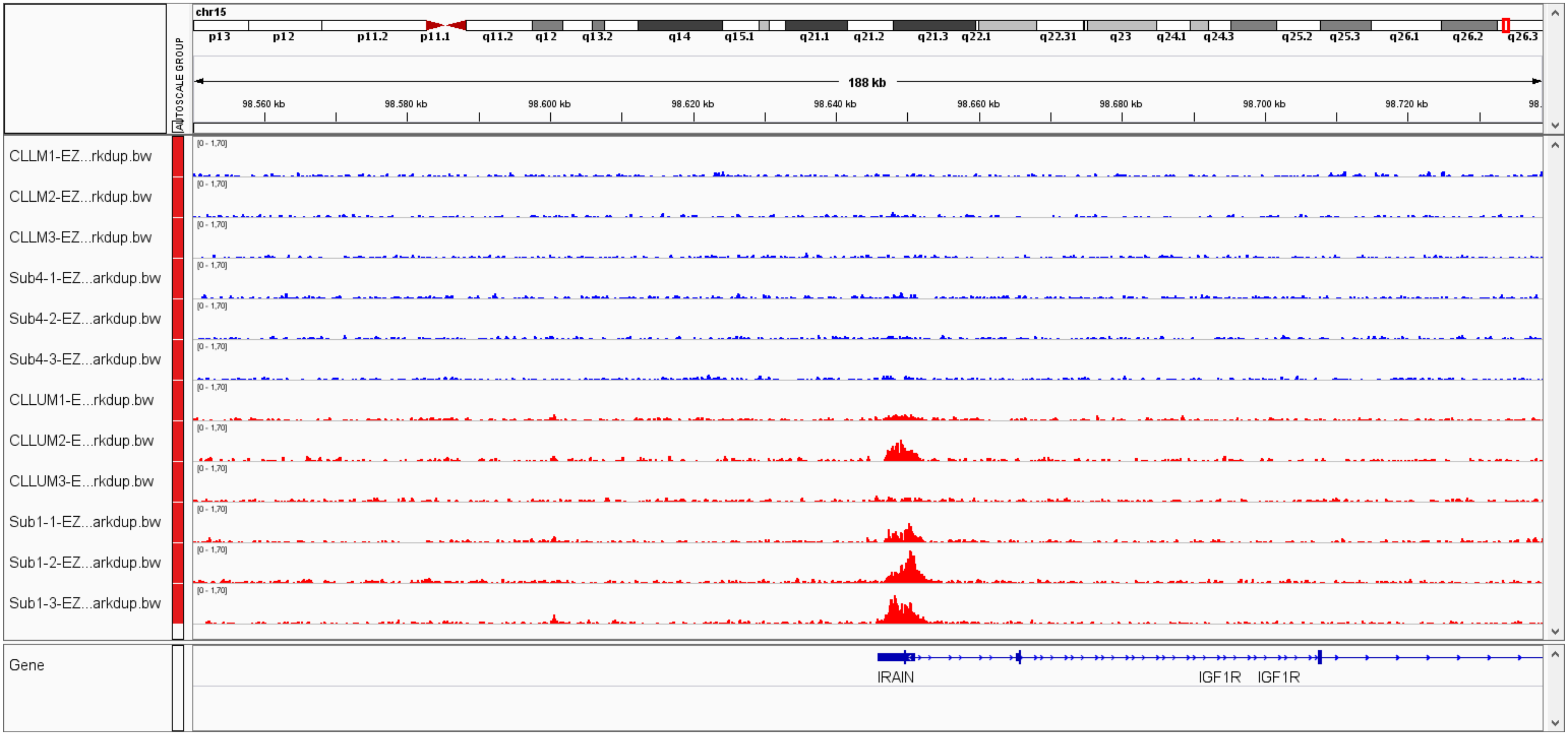

Figure S4: Differential binding of EZH2 methyltransferase to *IGF1R* promoter region in Unmutated CLL (U-CLL) subgroup (signal shown in red) compared to Mutated CLL (M-CLL) subgroup (signal shown in blue). The statistically significant (FDR-adjusted p.value < 0.001), differential binding of EZH2 was detected through DiffBind analysis during the CWL-based ChIP-Seq pipeline execution. Visualization was produced by IGV [1], using bigwig files (BW) that were produced using deeptools2 [2].

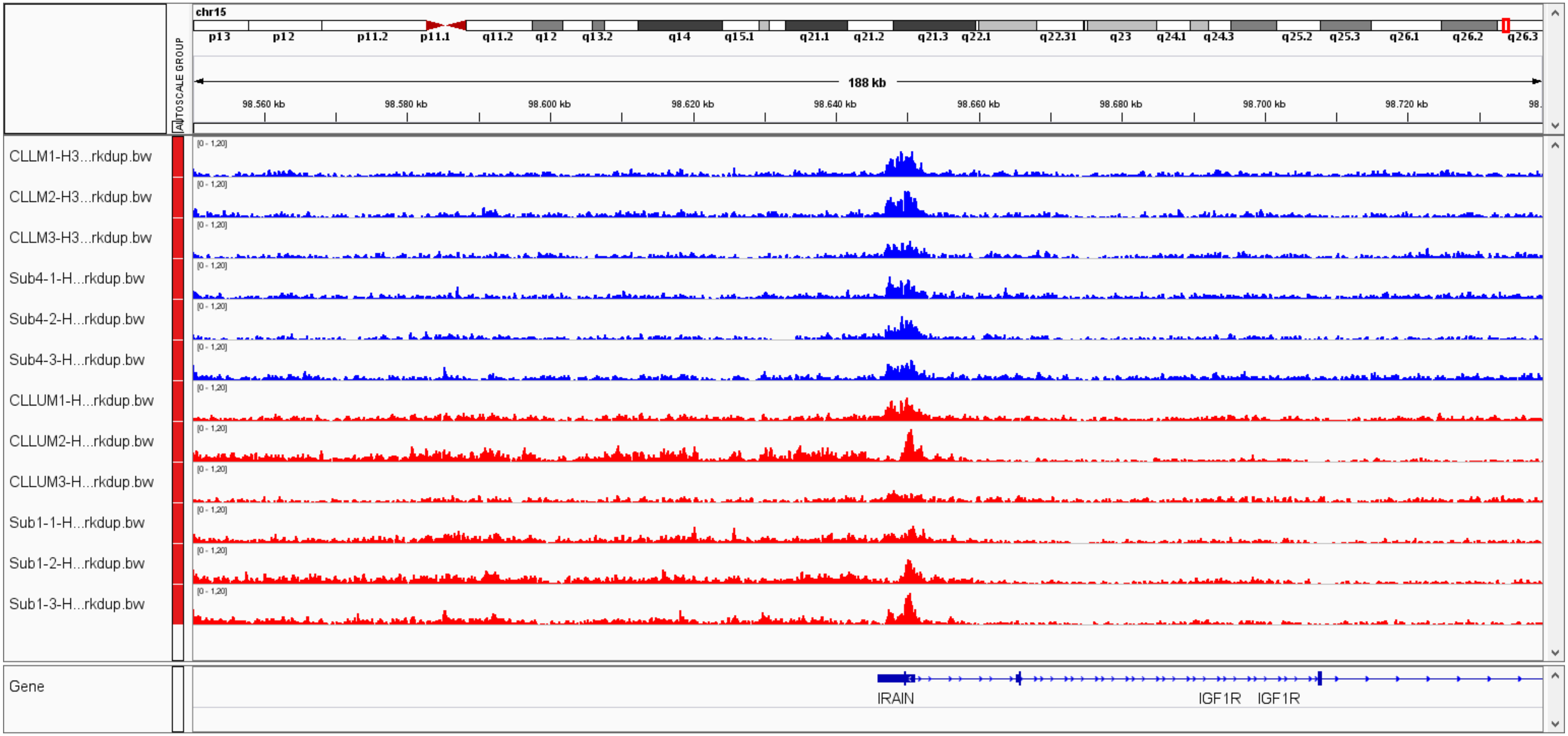

Figure S5: H3K27me3 signal detection across the IGF1R promoter region in Unmutated CLL (U-CLL) (signal shown in red) and Mutated CLL (M-CLL) (signal shown in blue) subgroups. No statistically significant, differential binding events were detected for the H3K27me3 modification, between U- and M-CLL subgroups, during the CWL-based ChIP-Seq pipeline execution. Visualization was produced by IGV [1], using bigwig files (BW) that were produced using deeptools2 [2].

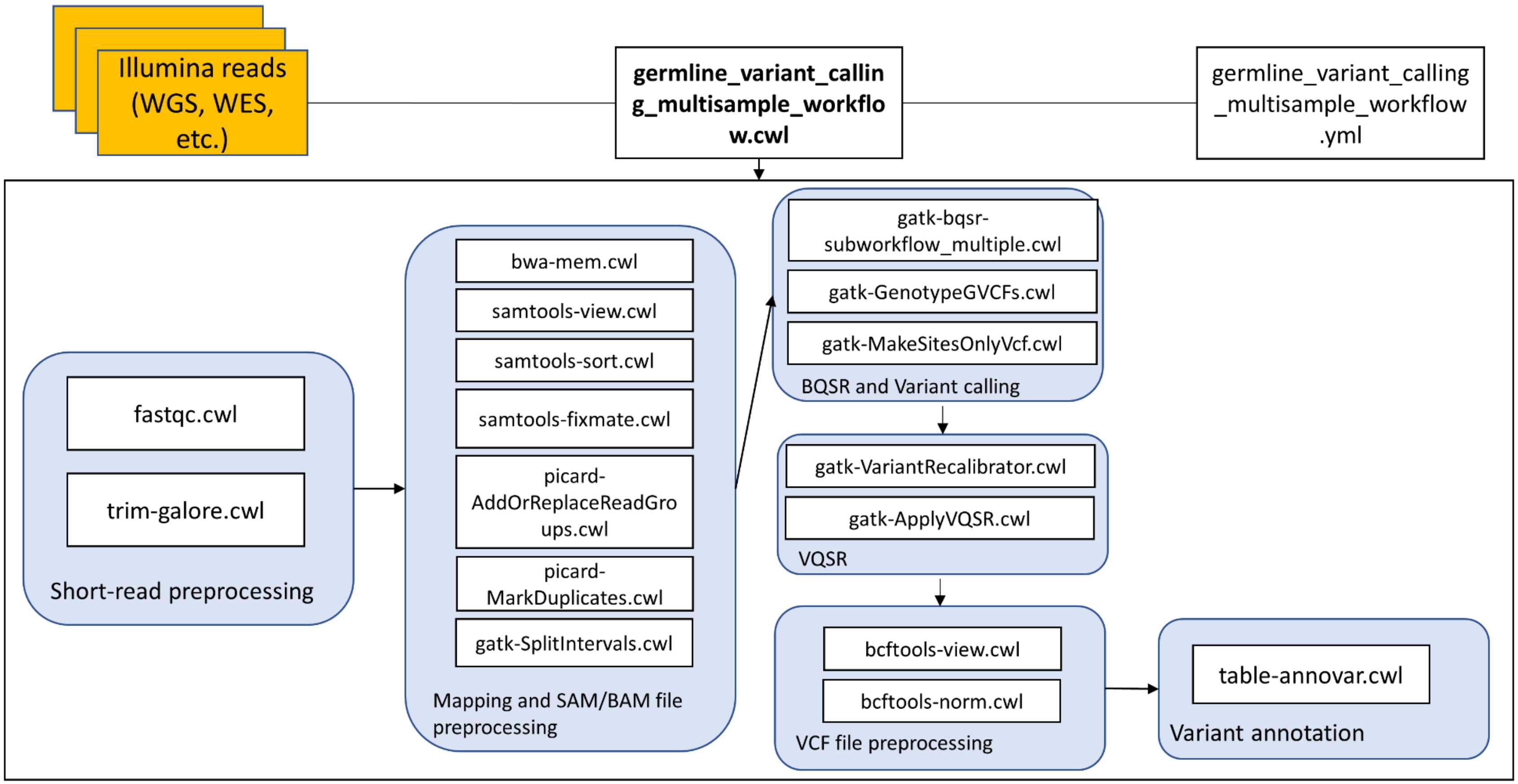

Figure S6: Diagram illustrating the main steps of the automated, CWL-based germline variant calling pipeline (multi-sample workflow) designed for the analysis of short-read data (WGS, WES, etc.). Briefly, the workflow integrates quality control (QC), trimming, mapping and preprocessing steps with the goal of performing germline variant calling, filtering and annotation. The workflow follows the approach of concatenating multiple samples into a single, unified VCF during the GATK HaplotypeCaller step (GVCF mode). The code for the workflow is available in the respective GitHub repository. Several steps of CWL ExpressionTools performing tasks such as moving/copying file(s) were excluded from the diagram for visualization purposes.

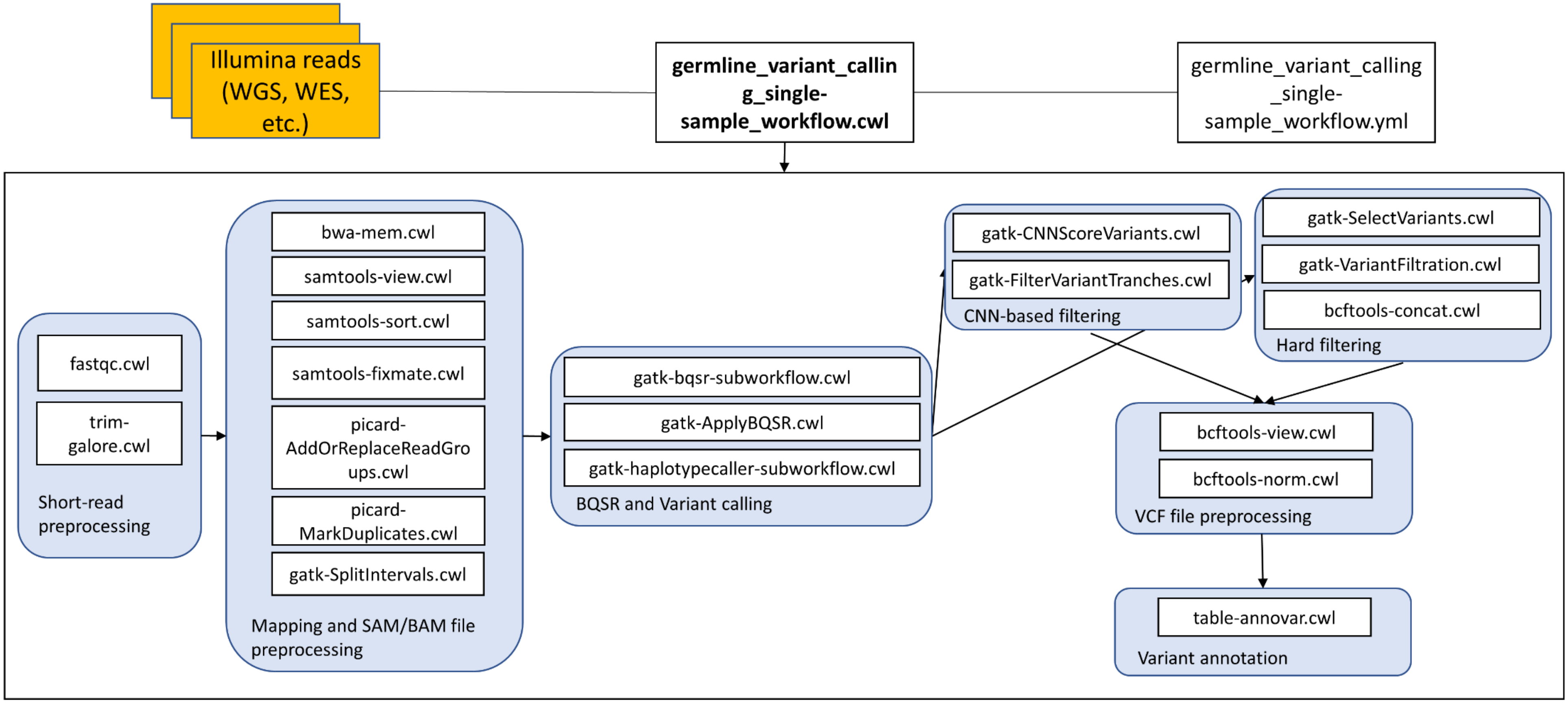

COMMON WORKFLOW LANGUAGE

docker

Figure S7: Diagram illustrating the main steps of the automated, CWL-based germline variant calling pipeline (single-sample workflow) designed for the analysis of short-read data (WGS, WES, etc.). Briefly, the workflow integrates quality control (QC), trimming, mapping and preprocessing steps with the goal of performing germline variant calling, filtering and annotation. Rather than merging the results of all samples into a unified VCF file, they are processed separately in each step of the workflow, leading to the production of a VCF file for each of them. The code for the workflow is available in the respective GitHub repository. Several steps of CWL ExpressionTools performing tasks such as moving/copying file(s) were excluded from the diagram for visualization purposes.

References

1. Papakonstantinou N, Ntoufa S, Tsagiopoulou M, Moysiadis T, Bhoi S, Malousi A, et al.. Integrated epigenomic and transcriptomic analysis reveals TP63 as a novel player in clinically aggressive chronic lymphocytic leukemia. Int J Cancer. 2019; doi: 10.1002/ijc.31999.

2. Kyritsis KA, Pechlivanis N, Psomopoulos F. Supporting data for “Software pipelines for RNA-Seq, ChIP-Seq and Germline Variant calling analyses in Common Workflow Language (CWL).” Zenodo; doi: 10.5281/zenodo.8383276

3. Robinson JT, Thorvaldsdóttir H, Winckler W, Guttman M, Lander ES, Getz G, et al.. Integrative genomics viewer. Nature Biotechnology. 2011; doi: 10.1038/nbt.1754.

4. Ramírez F, Ryan DP, Grüning B, Bhardwaj V, Kilpert F, Richter AS, et al.. deepTools2: a next generation web server for deep-sequencing data analysis. Nucleic Acids Res. 2016; doi: 10.1093/nar/gkw257.
